# Atomic Force Microscopy Analysis of Progenitor Corneal Epithelial Cells Fractionated by a Rapid Centrifugation Isolation Technique

**DOI:** 10.1371/journal.pone.0059282

**Published:** 2013-03-26

**Authors:** Wei Zhang, Zongyin Gao, Dongping Shao, Liu Zhang, Caixia Wang, Yuping Zhang

**Affiliations:** 1 Department of Ophthalmology, Guangzhou First People's Hospital, Guangzhou Medical University, Guangzhou, P. R. China; 2 Department of Ophthalmology, the Affiliated Nanhai Hospital of Southern Medical University, Foshan, Guangdong Province, P. R. China; 3 Department of Hematology, Guangzhou First People's Hospital, Guangzhou Medical University, Guangzhou, P. R. China; Dalhousie University, Canada

## Abstract

**Purpose:**

To investigate the use of atomic force microscopy (AFM) to image the three groups of corneal epithelial cells fractionated by a novel rapid centrifugation isolation technique.

**Methods:**

Epithelial cells harvested from primary cultures of rabbit limbal rings were centrifuged onto uncoated dishes, first at 1400 rpm and then at 1800 rpm. The adherent cells after centrifugation at 1400 rpm (ATC1), the adherent cells at 1800 rpm (ATC2) and the non-adherent cells at 1800 rpm (NAC) were investigated for BrdU retention and were subjected to contact mode AFM and Transmission Electron Microscopy (TEM).

**Results:**

Compared with unfractionated cells, the ATC1 group, accounting for about 10% of the whole population, was enriched in BrdU label-retaining cells. There were dramatic overall shape, surface membrane and intra-cellular ultrastructure differences noted among ATC1, ATC2 and NAC populations. The whole cell roughness measurements were 21.1±1.5 nm, 79.5±3.4 nm and 103±4.6 nm for the ATC1, ATC2 and NAC groups, respectively. The mero-nucleus roughness measurements were 34.2±1.7 nm, 13.0±0.8 nm and 8.5±0.5 nm in the ATC1, ATC2 and NAC populations, respectively.

**Conclusions:**

AFM was found to be a good tool for distinguishing among the three groups of cells. BrdU label retention, the AFM parameters and TEM together suggest that the ATC1, ATC2 and NAC populations may be progenitor corneal epithelial cells, transit amplifying cells and terminal differentiation cells, respectively.

## Introduction

Atomic force microscopy (AFM) is a powerful technique established as a surface science method that is capable of investigating material surfaces from near atomic resolution to mesoscales. [1] AFM allows for the noninvasive examination of specimens under natural conditions and with minimal preparation, and also enables the imaging of living cells in vitro and in vivo. [2] The greatest advantage of atomic force microscopy is its ability to obtain topographic information from the surface of a specimen in nonaqueous, aqueous, or dry conditions without staining, coating or freezing. [3] This allows for the observation of the specimen in conditions close to its natural environment. Only a few reports have been published on the application of AFM to observe the corneal epithelium and corneal epithelial cells. Marco Lombardo et al. [4] showed that AFM is capable of imaging and analyzing the corneal epithelium and the photoablated corneal stroma. In this experiment, AFM proved to be a high-resolution imaging tool for the scanning of both native as well as photoablated corneal specimens, and it permits precise topographic analysis of the corneal plane on the nanoscale. Kumar Sinniah et al. [5] investigated the use of AFM to image live and fixed cells in culture. Rabbit corneal fibroblasts, Chang conjunctival cells, and transformed human corneal epithelial cells were studied by AFM. These authors found that atomic force microscopy can be used to study cells and provide sub-cellular details at a resolution equal to or in some situations better than the scanning electron microscopy technique. Tsilimbaris et al. [6] evaluated the feasibility of imaging the normal corneal epithelium by means of AFM. Their work defined the AFM parameters appropriate for corneal epithelium imaging in a physiological medium. They concluded that AFM represents a new powerful tool for corneal epithelium imaging, and its application in this field warrants further investigation.

Corneal epithelial cells are classified as three types of cells: stem cells, transient amplifying cells, and terminally differentiated cells. [7] The corneal surface is renewed during healing after injury by cells that migrate from the limbus. These cells originate from limbal stem cells that reside in the basal layer of the limbus and represent a minor fraction of a heterogeneous limbal cell population. When total limbal stem cell deficiency (LSCD) occurs, it can be successfully treated by an allograft or autologous limbal cell transplantation. [8] As allogeneic and autologous cell sources for transplantation are limited, tissue engineering has evolved as one of the most promising therapies in regenerative medicine. [9] Optimal cell sources are very important. Isolated or at least enriched limbal SCs from the heterogenous population of limbal epithelial cells could enable the construction of regenerating corneal surfaces with normal phenotypes and improve our understanding of the characteristics of corneal epithelial stem cells. The lack of a definitive or unique biological marker introduces a degree of uncertainty to the unequivocal isolation and characterization of limbal stem cells. Some approaches have attempted to isolate stem cells from limbal cell cultures based on their characteristics, mainly the SP phenotype [10], small cell size [11], slow cell cycle [12], cell clone morphology [13], and in vitro adhesion assays [14]. We previously developed a centrifugal cell seeding method for rapid and efficient reconstruction of the rabbit ocular surface with limbal stem cell deficiency (LSCD) in rabbits. [15] The human corneal epithelial cell line (SDHCEC1) was used as the cell source. At 1800 rpm, 80% of cells can be attached to the uncoated dish within a short seeding time of 4 minutes, with 20% of cells remaining suspended in the medium. For cultured rabbit corneal limbal epithelial cells, only 20% of cells can attach to the uncoated dish at the same centrifugation force and time, while 80% of cells remain suspended in the medium. Why are these results different between the human cell line and cultured rabbit cells? SDHCEC1 cells were demonstrated to have stem cell characteristics, and the cells that become attached using the centrifugation method were found to have the greatest viability. Perhaps centrifugation fractions corneal epithelial stem cells from cultured cells. To address this, the cells were subjected to different centrifugal speeds, revealing three populations of corneal epithelial cells with different characteristics. The cells were studied by AFM, a non-destructive and high magnification microscopic method that provides detailed information on the microstructure of the corneal epithelial cells. AFM proved to be a feasible and accurate method of imaging the three-dimensional (3D) sub-microscopic cellular structure and also provided quantitative information on the cellular surface topography, enabling a comparison between the three groups of corneal epithelial cells.

## Materials and Methods

### Primary Explant Cultures of Rabbit Limbal Epithelial Cells

New England white rabbits were purchased from the Animal Laboratory of Guangzhou Medical University (Guangzhou, China). All procedures were performed according to the ARVO statement of the use of animals in ophthalmic and visual research. The protocol was approved by the Committee on the Ethics of Animal Experiments of Guangzhou Medical University (Permit Number: SCXK 2008–0002). All surgery was performed under sodium pentobarbital anesthesia, and all efforts were made to minimize suffering.

Primary cell culture was performed using the explant culture method. Briefly, the limbal rim was cut into pieces of approximately 2×2 mm. Two pieces were placed with the epithelium side down directly into six well culture plates and were left uncovered for 20 min before the culture medium was supplemented. The explants were then cultured in Dulbecco’s modified Eagle’s medium (DMEM; Gibco BRL) supplemented with 10 ng/mL human epidermal growth factor (EGF; Gibco BRL), 15% heat inactivated fetal bovine serum (FBS; Gibco BRL), 5 mg/mL human transferrin (Sigma), 5 mg/mL insulin, 0.4 mg/mL hydrocortisone (Gibco BRL), 100 U/mL penicillin, 100 mg/mL streptomycin (HyClone), and 2 mM L-glutamine at 37°C under 5% CO2 and 95% humidity. The medium was renewed every 2 days. The sub-confluent primary cultures were trypsinized on days 12–18 to prepare single cell suspensions for isolation of putative stem cells.

### Isolation of Limbal Epithelial Cells Using Centrifugal Force Adhesion

Rabbit limbal epithelial cells isolated from primary cultures were used for putative stem cell enrichment using a centrifugation method. The centrifugal seeding was carried out in an Eppendorf 5810R centrifuge with a rotor that had a radius of 20 cm. First, all single cells were suspended in the medium at a density of 9×10^5^ cm^−2^ and then centrifugally isolated at 1400 rpm for 4 min in uncoated six well culture plates. Attached cells (ATC1) and floating cells were collected, and the cells that remained unattached following centrifugation at 1400 rpm were then centrifuged at 1800 rpm for 4 min. Attached cells (ATC2) and floating cells (NAC) after centrifugation at 1800 rpm were then collected. Unfractionated cells that were not subjected to centrifugal adhesion separation were used as a control.

### Enrichment for BrdU Label-retaining Cells

Cells were labeled with BrdU and analyzed by a previously reported method [16] with modifications. Explant cultures in dishes were incubated with medium containing 10 µM BrdU when cellular outgrowth reached a 2–3 mm diameter, typically at day 3–5. The cultures were switched to BrdU-free medium after 72 h BrdU labeling, cultured for an additional 18 days, and then used for fractionation experiments as described above. The four groups of cells were fixed in cold methanol at 4°C for 10 min and processed for immunofluorescent staining with a BrdU antibody.

### Single Cell AFM Measurement

We collected three groups of cell samples: group 1, ATC1; group 2, ATC2; and group 3, NAC. The cells were passed to 6-well plates containing cover slips and cultured for 24 hours, at which time a phase-contrast micrograph was captured. The cells on the cover slips were then washed in phosphate buffered saline solution (pH 7.4), fixed with 1% glutaraldehyde for 10 min and then dried in air for AFM scanning.

Topographic images of cells were obtained using an AFM (Autoprobe CP Research, Veeco, USA) in contact mode. In all AFM experiments, gold-coated silicon nitride tips (UL20B, Park Scientific Instruments) with a spring constant of 2.5 N/m and a tip diameter of 20 nm were used. An optical microscope was used to help select the desired cells and direct the position of the AFM tip. Single-cell imaging was performed for ten cells from each condition, and each cell was scanned three times. To gain information on the topography of the cells, all images were analyzed using the instrument-equipped IP 2.1 software. The area analyzed with AFM was 100 µm×100 µm square. Ra defines the average surface fluctuation of the epithelial cells. All of these parameters were directly determined using the IP2.1 software.

### Transmission Electron Microscopy

We collected three groups of cell samples: group 1, ATC1; group 2, ATC2; and group 3, NAC. The three groups were fixed in 2.5% glutaraldehyde in 0.1 M phosphate buffer (pH 7.4), postfixed in 0.1 M osmium tetroxide, embedded in resin, and cut in 60-nm sections. The thin sections were observed and photographed under a transmission electron microscope (H600; Hitachi, Ltd., Tokyo, Japan).

### Statistical Analysis

All values were expressed as mean ± standard error. All statistical analyses were performed with SPSS software version 13.0. To study the morphologic parameters among the three groups, data were analyzed using a one-way analysis of variance (ANOVA). Statistical significance was defined as P-values <0.05.

## Results

### Centrifugal Force Adhesion Properties of Limbal Epithelial Cells

**Figure 1:**
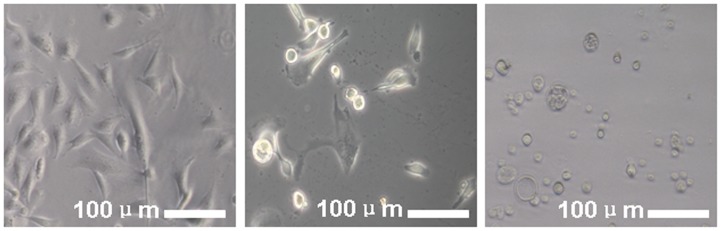
Optical microscopy images of the ATC1, ATC2 and NAC cells showed dramatically different morphologies among the three groups after fractionation by centrifugation and culture in medium for 1 day. Scale bar = 100****µm.

As shown in Fig. 1, the three groups of cells exhibited dramatically different morphologies after culture. The ATC1 group grew well and showed a more mosaic epithelium with a homogeneous morphology. The ATC2 group increased in size with drawing-off and exhibited a heterogeneous morphology. The NAC group exhibited rounded, aging appearance with vacuoles, with some of the cells detached from the dishes and floating in the medium.

### ATC1 Cells are Enriched for BrdU Label-retaining Cells

**Figure 2:**
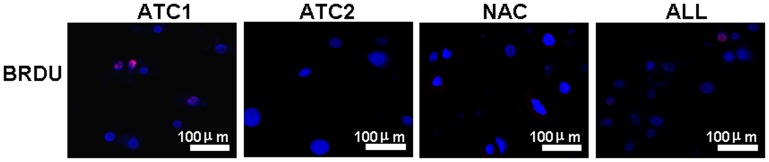
Representative immunofluorescent staining for BrdU label-retaining cells in four populations of limbal epithelial cells: three populations selected by centrifugation adhesion to uncoated dishes (ATC1, ATC2 and NAC) and all unfractionated cells (ALL). Centrifugation adhesion experiments were performed after the cell cultures were labeled with 10 µm BrdU for 72 hr and then chased in the absence of BrdU for 18 days. Scale bar = 100****µm.

As shown in Fig. 2, after 72 hr labeling with BrdU followed by a chase for 18 days, the ATC1 population contained the highest number of label-retaining cells (LRCs). The ATC1 group had a higher number of LRCs than the unfractioned whole cell population, but the ATC2 and NAC showed almost no LRCs.

### AFM Analysis

#### Analysis of whole cell samples

**Figure 3:**
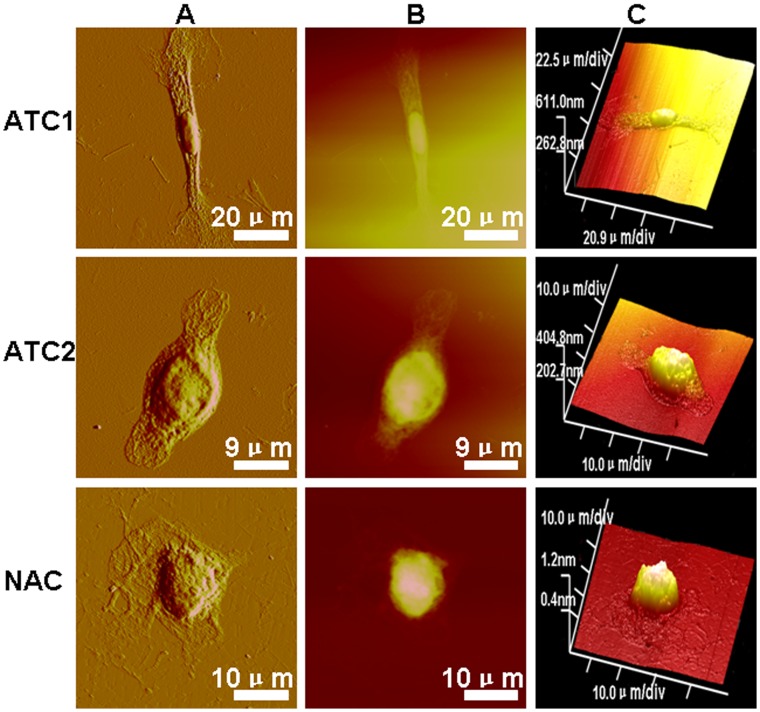
Representative AFM topographic data of single ATC1, ATC2 and NAC cells (A: peak force error; B: height sensor; C: 3D image) showed dramatically different morphologies among the three groups. Scanning area: ATC1∶100×100 µm; ATC2∶45×45 µm; NAC: 50×50 µm.

**Figure 4:**
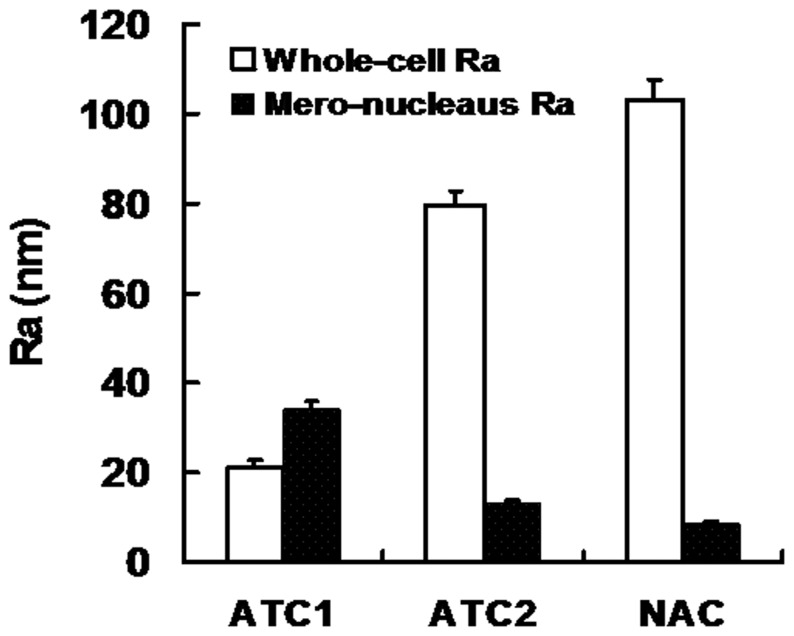
Surface ultrastructures of ATC1, ATC2 and NAC cells (A: peak force error; B: 3D image) showed that the cell surface morphology was dramatically different between the three groups. Scanning area: 5×5 µm.

When whole cell samples were interrogated at low magnifications, AFM examinations revealed three different types of corneal epithelial cells. As shown in Fig. 3, ATC1 were typically polygonal and smooth epithelial cells. We were able to image the edges of migrating corneal epithelial cell membranes demonstrating abundant filopodias. The nucleus was very clear and sharply distinct from the cytoplasm. The whole cell roughness measurements were 21.1±1.5 nm (Fig. 4) (p<0.05). ATC2 cells exhibited a fusiform shape and appeared as moderately rough epithelial cells. The filopodias on the cells were less abundant and shorter compared to those on ATC1 cells. The nucleus was not so clear and sharply distinct from the cytoplasm as compared to ATC1. The whole cell roughness measurements were 79.5±3.4 nm (Fig. 4) (p<0.05). NAC cells exhibited a round shape and a highly rough epithelial cell surface. The nucleus could not be distinguished from the cytoplasm. The whole cell roughness measurements were 103±4.6 nm (Fig. 4) (p<0.05). Based on their surface appearance, we could distinguish at least three types of corneal epithelial cells with smooth, moderately rough, and rough surfaces.

#### Analysis of the mero-nucleus membrane

**Figure 5:**
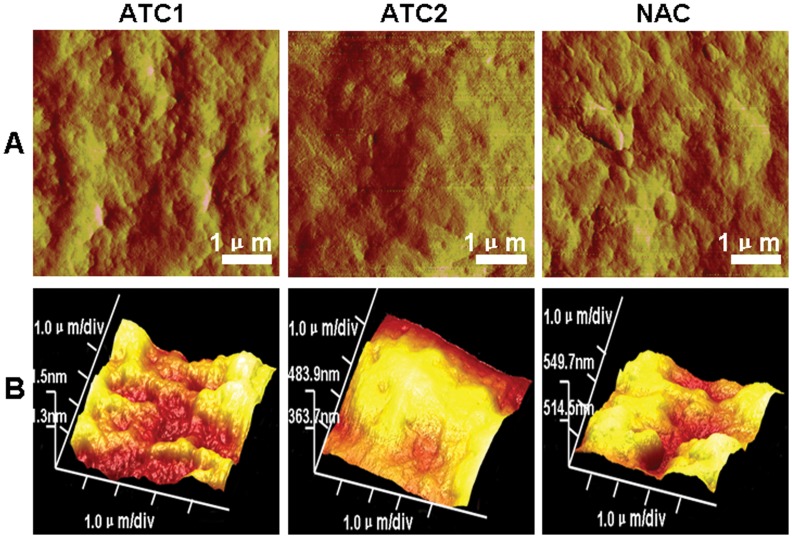
Histogram of roughness values of ATC1, ATC2 and NAC cells showed different tendencies between whole cell samples (p<0.05) and the mero-nucleus membrane (p<0.05).

AFM examination of the mero-nuclear membrane at high magnification revealed three different types of membrane images. As shown in Fig. 5, all cells display rounding protruding particles, namely microprojections, which we attribute to the cell nucleus. The loading force applied by the tip dents the surface of the cell enabling cytoskeletal features to be observed. Craterlike structures could be detected on the cell surface. The ATC1 membrane showed numerous densities of short protruding particles and numerous craterlike structures. The corresponding ultrastructures indicated a composite of membrane proteins with a regular nanoscale network. The mero-nucleus roughness measurements were 34.2±1.7 nm (Fig. 4) (p<0.05). The ATC2 membrane produced a smooth image, with a low density of shorter protruding particles and few craterlike structures as compared to ATC1. The ultrastructures indicated a composite of membrane proteins with an irregular nanoscale network. The mero-nuclear roughness measurements were 13.0±0.8 nm (Fig. 4) (p<0.05). The NAC membrane showed a variable density of irregular protruding particles and numerous craterlike structures. Larger particles were interspersed on the surface, implying a significant aggregation of membrane proteins. The mero-nucleus roughness measurements were 8.5±0.5 nm (Fig. 4) (p<0.05).

### A New Look at Limbal Epithelial Cells Intra-cellular Ultrastructure

**Figure 6:**
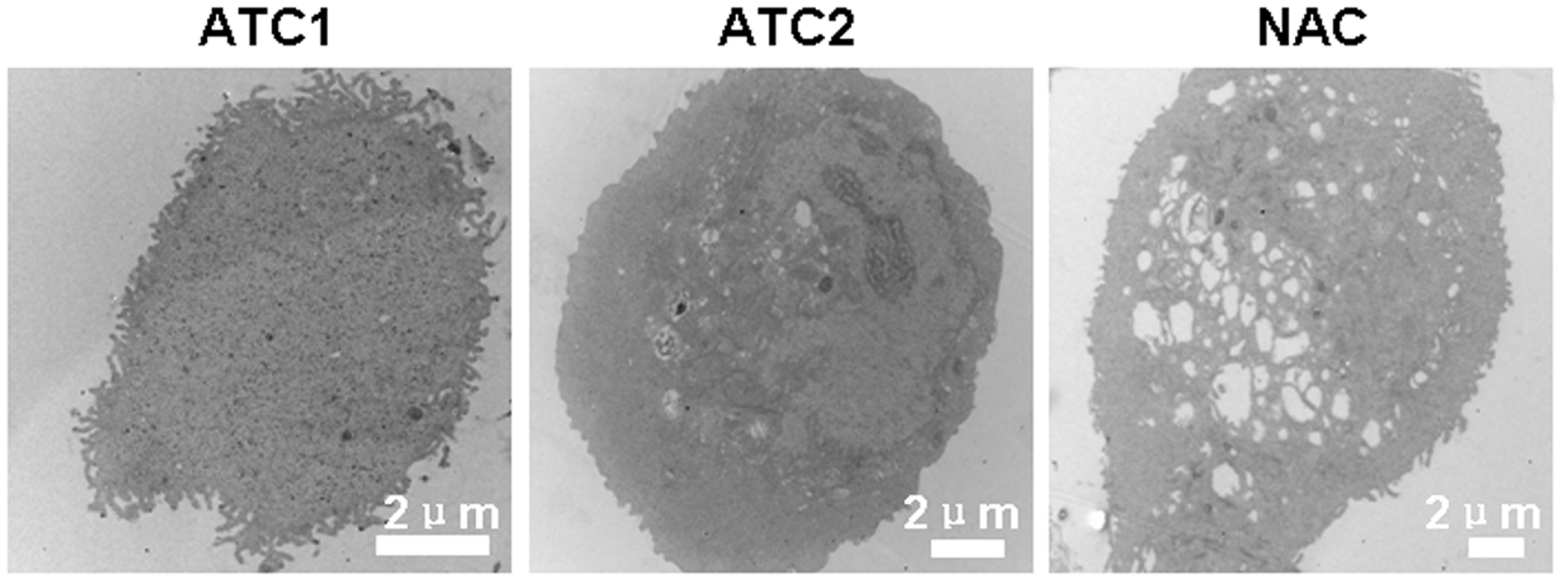
Representative images of transmission electron microscopy showed the ultrastructure of ATC1, ATC2 and NAC. (A) In the ATC1 group, there were many of microvilli and barely detectable nucleolus. (B) ATC2 group exhibited pronounced nucleolus, disruption of the microvilli and swelling of mitochondria. (C) NAC group showed the biggest size and the most swelling of mitochondria. Scale bar = 2****µm.

Transmission electron microscopy shows that ATC1 are the smallest size with the largest N/C ratio compare to ATC2 and NAC. Their nucleus has barely detectable nucleolus. With TEM, it could be seen that ATC1 displayed dense microvilli on their cell surface membrane (Fig. 6A). The ATC2 have an intermediate size between ATC1 and NAC with a low nucleus/cytoplasm (N/C) ratio. The nucleus has a pronounced nucleolus. In the ATC2 group, reduction of number and size of microvilli and swelling of mitomitochondria were observed (Fig. 6B). The NAC are the biggest size in the three groups with the lowest nucleus/cytoplasm (N/C) ratio. In the NAC group, reduction of number and size of microvilli and the most swelling of mitomitochondria in the three groups were observed (Fig. 6C).

## Discussion

This work has shown that corneal epithelial stem cells, transient amplifying cells and terminally differentiated cells can be selected using a centrifugation method. Furthermore, it is feasible to observe and distinguish the appearance and morphology of the three groups using AFM. We had been tried to obtain the images of biological samples under air or liquid conditions using AFM. While, the resolution of images obtained under ambient conditions is much higher than that of under liquid condition.

Although several stem cell markers have been proposed, specific biochemical markers have not been defined for identifying epithelial stem cells. Therefore, one of the valued methods of identification of stem cells relies on identifying slow-cycling cells using the BrdU label retaining method in vivo. [17] One known characteristic of epithelial stem cells in vivo is that these cells are slow cycling and retain BrdU labels over a long period.

In this study, we labeled the cultures continuously for 72 hr at their early growth stage (3∼5 days). At first, BrdU was incorporated into the DNA during the S phase in all mitotic cells, including the stem cells and transient amplifying cells. For longer labeling periods, more rapidly-cycling cells incorporated the label faster, matured, and died, while the BrdU-labeled stem cells still remained. When the cells were centrifuged, the ATC1 population contained the highest number of label-retaining cells (LRCs). The ATC1 group had a higher number of LRCs than that of the unfractioned whole cell population, but the ATC2 and NAC showed almost no LRCs. Based on this result, ATC1 cells collected at 1400 rpm were thought to be corneal epithelial stem cells. ATC2 and NAC cells were thought to be transient amplifying cells and terminally differentiated cells, respectively. De-Quan Li et al. [7] also attempted to isolate a population of human limbal epithelial cells enriched for certain putative stem cell properties based on their phenotype. Epithelial cells harvested from fresh human limbal rings and their primary cultures were allowed to adhere to collagen IV-coated dishes for 20 min and 2 hr, sequentially. The rapidly adherent cells (RAC), slowly adherent cells and non-adherent cells were evaluated for certain stem cell properties such as BrdU-label retention. They demonstrated that human limbal epithelial cells with stem cell properties can be partially enriched by their adhesiveness to collagen IV. Those results agree well with our findings, but our method is relatively simple and does not require collagen IV-coated dishes.

The AFM study of the three groups demonstrates that the method truly separates three different populations of corneal epithelia cells. In the whole cell sample AFM images, the ATC1 cells appeared to be smooth, with numerous filopodias and distinct nuclei. ATC1 cells exhibited the least whole cell roughness. The filopodias were less prevalent and shorter on the ATC2 cells, the roughness was moderate and the nucleus was not as distinct. NAC cells exhibited a round shape and appeared to be highly rough epithelial cells. The nucleus could not be distinguished from the cytoplasm. Tsilimbaris et al. studied [6] the surface roughness of the normal corneal epithelium of albino rabbits. Based on the roughness these authors were able to demonstrate the existence of three cell types with smooth, moderately rough, and rough surfaces. This categorization is in accordance with our AFM and Versura^’^s SEM findings [18], where three different apical epithelial cell types (dark, medium, and light) have also been described based on cells’ brightness. These SEM differences have been correlated with variations in the density of surface microplicae and with the cell’s age: light cells are considered young, whereas dark cells are thought to be old. One possible correlation could be that light, medium, and dark cells as observed by SEM correspond to smooth, moderately rough and rough cells, respectively, as observed by AFM. Brewitt et al. [19] showed that during wound healing the migrating cells have smooth surfaces, while after healing and stratification they show a rough surface. Therefore, the different ultra-structural characteristics of the epithelial cells may be related to the cell’s age. Tsilimbaris et al. [6] analyzed the roughness of the porcine epithelial cell surface and demonstrated that it was similar to that of the rabbit cornea. Further, in previous experiments Doughty [20] and Brewitt [19] showed that the human epithelial cell surface exhibits similar morphological features. These results also coincide with BrdU label-retaining experiments in which ATC1, ATC2 and NAC were considered to be corneal epithelial stem cells, transient amplifying cells, and terminally differentiated cells, respectively. Corneal epithelial stem cells are known to be young cells and have a greater nucleus to cytoplasm (N/C) ratio compared with the other two groups.

AFM examination revealed three different images of the mero-nucleus membrane. The cell membrane architecture of ATC1 cells was homogeneous while ATC2 and NAC cells feature a heterogeneous architecture. The ATC1 membrane showed numerous densities of short protruding particles and numerous craterlike structures, and the ultrastructure indicated a regular nanoscale network membrane protein. The mero-nuclear roughness was the greatest for ATC1 cells. The ATC2 membrane showed smooth images with few protruding particles or craterlike structures. The ultrastructure indicated an irregular nanoscale network of membrane proteins. The mero-nucleus roughness was moderate. The NAC membrane had numerous long and short irregular protruding particles and numerous craterlike structures. The ultrastructure also indicated an irregular nanoscale network of membrane proteins. Larger particles were interspersed on the surface of NAC membranes implying significant aggregation of membrane proteins. The mero-nuclear roughness measurements were the lowest for NAC cells. These structures may represent the appearance of the molecular structure of the epithelial cell membrane. From ATC1 to ATC2 and NAC cells, the ultrastructure of the cell surface changed from a homogeneous to a heterogenous morphology and the prevalence of microprojections, craterlike structures and Ra decreased.

Ojeda et al. [21] concluded that one of the functions of the microprojections is to increase cell surface area and therefore facilitate intra- and extracellular motion of molecules such as nutritional and waste products across the cell membranes. These conclusions agree with our results, ATC1 stem cells had the greatest vitality among the three groups, and they also had the greatest number of microprojections and craterlike structures with which to exchange nutritional and waste products across the cell membranes to maintain the metabolic rate. ATC2 cells were old age cells with a reduced metabolism and also fewer microprojections and craterlike structures. NAC cells showed more irregular microprojections and craterlike structures compared to ATC2, perhaps because these are terminally differentiated cells that will soon die. These conclusions from AFM also agree with the results from transmission electron microscopy. The three groups also appeared remarkably different under TEM revealed an obvious decrease of N/C ratio and microvilli and increase swelling of mitochondria from ATC1 to ATC2 and NAC cells. Kumar Sinniah et al. [5] showed that the loading force (0.5–1.0 nN) applied by the tip dents the surface of the cell enabling cytoskeletal features to be observed. In our experiments, the Ra of the mero-nucleus follows an opposite sequence of ATC1, ATC2 and NAC compared to that of the whole cell Ra. This result may be due to the 2.5 nN loading force applied in the region of the nucleus, and the fact that both cytoskeletal and membrane features could be detected. The results of AFM and TEM are consistency with each other. TEM showed an obvious decrease of compact structure of the cytoskeleton from ATC1 to ATC2 and NAC cells. For the ATC1 stem cells the nucleus, other organelles, cytoskeleton, membrane channels and membrane proteins have the greatest activity compared with ATC2 and NAC.

### Conclusion

This experiment successfully separated corneal epithelial stem cells, transient amplifying cells and terminally differentiated cells by a centrifugation method. AFM represents a powerful new tool for the imaging and observation of corneal epithelial cells. This work defined the AFM imaging parameters appropriate for three types of cornea epithelia cells and obtained high quality images that could distinguish among the three groups. It became obvious that there is new, interesting information to be obtained with AFM that merits further investigation in other ophthalmic tissues in the future.
